# Compression from a retroperitoneal schwannoma presenting as a subepithelial lesion of the gastric fundus

**DOI:** 10.1055/a-2271-4028

**Published:** 2024-03-11

**Authors:** Xue-Mei Lin, Juan Liu, Chun-Hui Xi, Jun Wang, Guo-Dong Yang, Xian-Fei Wang, Cong Yuan

**Affiliations:** 1 Department of Pathology, Institute of Basic Medicine and Forensic Medicine, North Sichuan Medical College, Nanchong, China; 2 Department of Pathology, Affiliated Hospital of North Sichuan Medical College, Nanchong, China; 3 Department of Gastroenterology, Affiliated Hospital of North Sichuan Medical College, Nanchong, China; 4 Digestive Endoscopy Center, Affiliated Hospital of North Sichuan Medical College, Nanchong, China

Gastric subepithelial lesions (SELs) are frequently incidental findings encountered during endoscopy. The lesions may originate from any layer of the gastric wall and sometimes arise from compression by extraluminal structures 1 . Schwannomas are nerve sheath tumors that seldom occur in the retroperitoneal region, comprising only 4% of all retroperitoneal tumors and 3% of all schwannomas 2 . Herein, we present a case of retroperitoneal schwannoma compressing the gastric fundus and presenting as a protruding subepithelial mass.

A 66-year-old woman underwent esophagogastroduodenoscopy (EGD) for screening purposes. EGD revealed an SEL in the gastric fundus that protruded into the stomach cavity ( Fig. 1 **a** ). Endoscopic ultrasonography (EUS) showed that the subepithelial protrusion derived from extraluminal compression rather than an intramural lesion ( Fig. 1 **b** ; Video 1 ). Computed tomography scanning confirmed that the extraluminal compression was being caused by a nodular low-density mass between the abdominal aorta and the gastric wall, which was approximately 2.5 cm in size, with clear boundaries and uneven mild enhancement ( Fig. 1 **c** ). The patient refused EUS-guided fine-needle aspiration. Laparoscopic surgery revealed that the mass was located in the retroperitoneum, and it was removed. Pathological examinations subsequently revealed a schwannoma ( Fig. 2 ).

**Fig. 1:**
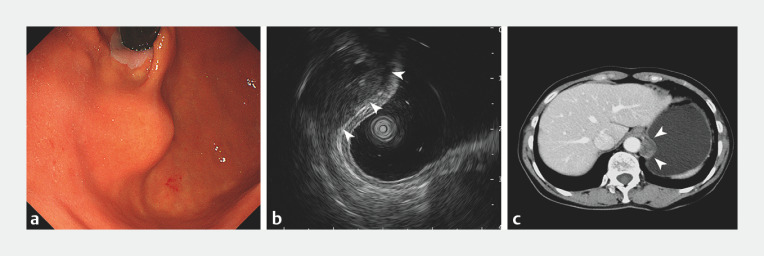
Endoscopic, endosonographic, and computed tomography (CT) findings of fundal submucosal lesions. **a** Endoscopic views showed a fundal protrusion with normal overlying mucosa, suggestive of a subepithelial lesion. **b** Endoscopic ultrasonography revealed an intact gastric wall covering the protrusion, indicating extraluminal compression arising from a heterogeneously hypoechoic structure adjacent to the wall (arrows). **c** CT scanning demonstrated a nodular mass of approximately 2.5 cm in diameter between the abdominal aorta and the gastric wall, which was causing gastric fundal compression (arrows).

**Fig. 2:**
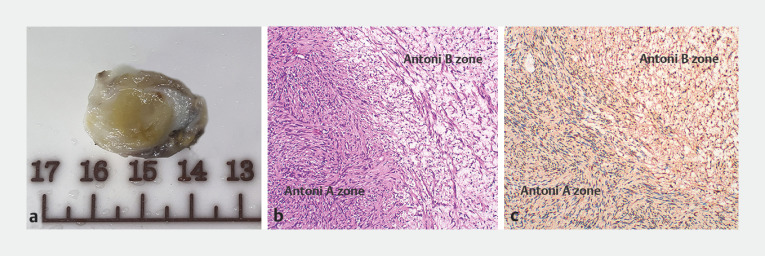
Histopathological examination. **a** Macroscopic appearance of the cut surface of the resected mass showed a yellow, firm, and encapsulated solid tumor. **b** Histopathologic findings revealed a dense arrangement of short spindle-shaped cells in a fasciculated and disarrayed architecture (Antoni A zone) alternating with sparsely arranged round or ovoid cells in scattered asterisms (Antoni B zone). No pathologic mitosis was observed (hematoxylin and eosin staining, ×100). **c** The tumor cells were diffuse positive for S-100 (immunohistochemical staining, ×100).

Gastroscopy showed a subepithelial lesion in the gastric fundus. Endoscopic ultrasonography and computed tomography demonstrated that the protrusion arose from extraluminal compression by an adjacent mass. Retroperitoneal schwannoma was confirmed postoperatively.Video 1

Common sources of extrinsic gastric compression are normal abdominal structures, such as the spleen, splenic vessels, gallbladder, colon, and pancreas 3 . In addition, pathologic conditions such as tumors, cysts, aneurysms, ectopic pancreas, and enlarged lymph nodes may appear as gastric SELs on endoscopy 4 . Extragastric compression may be difficult to distinguish from intramural lesions using endoscopy alone. In this situation, EUS can provide reliable information for the differentiation of extraluminal compression from true subepithelial tumors 3 . Although retroperitoneal schwannoma is a rare tumor, this case illustrates that the entity should also be included in the differential diagnosis of gastric SELs.

Endoscopy_UCTN_Code_CCL_1AB_2AD_3AB

## Notes

Endoscopy E-Videos https://eref.thieme.de/e-videos
*E-Videos* is an open access online section of the journal *Endoscopy* , reporting on interesting cases and new techniques in gastroenterological endoscopy. All papers include a high-quality video and are published with a Creative Commons CC-BY license. Endoscopy E-Videos qualify for HINARI discounts and waivers and eligibility is automatically checked during the submission process. We grant 100% waivers to articles whose corresponding authors are based in Group A countries and 50% waivers to those who are based in Group B countries as classified by Research4Life (see: https://www.research4life.org/access/eligibility/ ). This section has its own submission website at https://mc.manuscriptcentral.com/e-videos .
